# Quality of life impact of cardiovascular and affective conditions among older residents from urban and rural communities

**DOI:** 10.1186/1477-7525-11-140

**Published:** 2013-08-14

**Authors:** Joanne Allen, Kerry J Inder, Melissa L Harris, Terry J Lewin, John R Attia, Brian J Kelly

**Affiliations:** 1 Centre for Translational Neuroscience and Mental Health, University of Newcastle and Hunter New England Health, Newcastle, NSW, Australia; 2 Hunter Medical Research Institute, Newcastle, NSW, Australia; 3 Research Centre for Gender, Health and Ageing, Faculty of Health, University of Newcastle, Newcastle, NSW, Australia; 4 Centre for Clinical Epidemiology and Biostatistics, University of Newcastle and Hunter New England Health, Newcastle, NSW, Australia

**Keywords:** Cardiovascular disease, Urban–rural, Social capital, Quality of life, Physical and psychological health, Over 55 years

## Abstract

**Background:**

The demographic, health and contextual factors associated with quality of life impairment are investigated in older persons from New South Wales, Australia. We examine the impact of cardiovascular and affective conditions on impairment and the potential moderating influence of comorbidity and remoteness.

**Methods:**

Data from persons aged 55 and over were drawn from two community cohorts sampling from across urban to very remote areas. Hierarchical linear regressions were used to assess: 1) the impact of cardiovascular and affective conditions on physical and psychological quality of life impairment; and 2) any influence of remoteness on these effects (N = 4364). Remoteness was geocoded to participants at the postal code level. Secondary data sources were used to examine the social capital and health service accessibility correlates of remoteness.

**Results:**

Physical impairment was consistently associated with increased age, male gender, lower education, being unmarried, retirement, stroke, heart attack/angina, depression/anxiety, diabetes, hypertension, current obesity and low social support. Psychological impairment was consistently associated with lower age, being unmarried, stroke, heart attack/angina, depression/anxiety and low social support. Remoteness tended to be associated with lower psychological impairment, largely reflecting overall urban versus rural differences. The impacts of cardiovascular and affective conditions on quality of life were not influenced by remoteness. Social capital increased and health service accessibility decreased with remoteness, though no differences between outer-regional and remote/very remote areas were observed. Trends suggested that social capital was associated with lower psychological impairment and that the influence of cardiovascular conditions and social capital on psychological impairment was greater for persons with a history of affective conditions. The beneficial impact of social capital in reducing psychological impairment was more marked for those experiencing financial difficulty.

**Conclusions:**

Cardiovascular and affective conditions are key determinants of physical and psychological impairment. Persons affected by physical-psychological comorbidity experience greater psychological impairment. Social capital is associated with community remoteness and may ameliorate the psychological impairment associated with affective disorders and financial difficulties. The use of classifications of remoteness that are sensitive to social and health service accessibility determinants of health may better inform future investigations into the impact of context on quality of life outcomes.

## Introduction

The impact of geographic factors on health outcomes, particularly for persons affected by chronic health conditions, has long been of concern. Rural areas are often characterised by poor access to health services, increased risk of injury, and stress due to adverse environmental conditions and socioeconomic disadvantage [1-4]. However, rural areas typically display high levels of social capital that may be protective against poor health and functioning [5-7]. Social capital is a multidimensional term used to describe social interactions within communities that promote [8] and are embedded within [9] norms of trust, reciprocity and social cohesion, which support the actions of persons in these communities [10,11]. Social capital is thought to influence health both through psychosocial pathways that promote individual adaptation to adversity [12] and by enabling access to health-related resources (i.e. by facilitating the spread of health related information, opportunities and attitudes) [13]. There is increasing evidence that contextual effects, such as availability of health services, socio-economic deprivation [14,15], aggregate social capital [16-19] and remoteness [20-22] influence the experience of disease and health.

Current estimates suggest that over half of adults report at least one chronic physical health condition [23,24] and among individuals aged 65 and over, this figure is approximately 80% [25]. The 2007 Australian National Survey of Mental Health and Wellbeing revealed that 45.6% of people who met criteria for a 12-month affective or anxiety disorder also reported a current chronic physical health condition [26]. Such mental health conditions are known to exacerbate the disability associated with chronic physical conditions [27] and vice versa [28,29]. These findings suggest chronic physical health conditions are of significant concern, particularly for older persons and those with mental health conditions.

The burden associated with comorbid physical and mental health conditions was more recently raised by the 2012 National Report Card on Mental Health and Suicide Prevention [30], with specific reference to the burden of cardiovascular conditions in persons with a history of mental illness and those living in rural and remote areas of Australia. Consideration of the underlying mechanisms and ways to equitably address the issues of intervention and treatment of comorbid mental-physical conditions in these populations is ongoing [30,31]. However, social capital may be particularly relevant to intervention and understanding regarding the burden associated with cardiovascular conditions and depression, which have been linked to prolonged stress responses [32-34], socioeconomic disadvantage [35] and stressful life events [36,37]. Attention to the role of such contextual factors in health related quality of life (HRQoL) provides an opportunity to improve our understanding of a broad range of potential influences on the burden associated with physical and mental health conditions.

To date, rural and remote regions of Australia have been underrepresented in national surveys and little detailed information from these populations is available for comparison with urban populations. The Extending Treatments, Education and Networks in Depression (xTEND) study is a collaboration between two existing and ongoing longitudinal cohorts, namely the Australian Rural Mental Health Study (ARMHS) and Hunter Community Study (HCS), that aims to investigate the social determinants of wellbeing in Australia and how these may be influenced by contextual factors associated with increasing remoteness [38]. Combined, these cohorts provide a sample representative of the spectrum of urban to very remote communities, in largely contiguous local government areas. These studies share common baseline assessments of psychological distress and HRQoL outcomes, as well as demographic and health related determinants, such as life time diagnoses of several chronic illnesses and indices of health behaviour. In combining individual level data from these cohorts, due consideration has also been given to similarities and differences in recruitment, methodology, and assessment techniques and recent work has confirmed that the administered assessment of HRQoL impairment taps divergent aspects of physical and psychological impairment which are invariant across these samples [39]. While physical and psychological HRQoL outcomes have been shown to have different demographic and social determinants, little evidence currently exists regarding the differential impact of chronic disease and contextual factors on these health domains. Such information would have implications for understanding the burden associated with these disorders, as well as tailoring interventions and treatment in light of contextual factors.

Thus, the purpose of the current paper is threefold. Firstly, in the *primary analysis*, we use data from the xTEND collaboration to represent older persons from across urban to very remote areas of New South Wales (NSW), Australia, and adopt a multi-level framework to investigate the impact of cardiovascular and affective conditions, community remoteness and their interactions upon physical and psychological HRQoL impairment. We also model the additional impacts of personal characteristics known to influence the association between perceived health and health conditions, including demographic characteristics, such as gender, age and socioeconomic status [40,41], as well as other health risk factors, including smoking [41], obesity [42] and social support [43]. Secondly, we use community data from the NSW Adult Population Health Survey, in a *secondary analysis*, to examine an index of community remoteness in terms of its correspondence with self-reported health service accessibility and aspects of social capital. Finally, in a *sub-analysis* of the ARMHS data, we examine whether direct measures of social capital influenced outcomes of the primary analysis model and whether the influence of social capital moderates the influence of financial difficulty on HRQoL outcomes.

## Methods

### Participants

For the purposes of our *primary analysis*, self-report postal survey data from two NSW population-based cohort studies were combined: the HCS [44]; and the ARMHS [45]. Detailed descriptions of recruitment, sample descriptions and methods employed can be obtained from their respective baseline descriptive papers [HCS: 44, ARMHS: 45]. Briefly, the HCS is a study of persons aged 55–85 years residing in the major regional city of Newcastle and the ARMHS is a study of persons aged 18 years and older residing in non-metropolitan areas of NSW. Both studies randomly selected potential participants from the state electoral roll. Introduction and recruitment letters were sent to individuals by post and non-responding individuals were followed-up by telephone calls. Overall response rates of 44.5% and 27.3% for the HCS and ARMHS respectively were achieved, with both samples having comparable rates of uncontactable or excluded persons (HCS: 26.9%, ARMHS: 25.2%). Within the ARMHS sample, among those who were contactable and met study inclusion criteria, participation rates varied by age group (under 55 years: 25.4%; 55–70 years: 32.4%; over 70 years: 20.1%). A comparable pattern emerged within the HCS sample, with responders tending to be slightly younger than non-responders (66.3 vs. 68.6 years) [26]. To reduce participant burden, survey items were administered over two postal surveys in both cohorts.

Following ethical approval (Human Research Ethics Committees from the University of Newcastle and Hunter New England Area Health), baseline survey data from the HCS and ARMHS were combined. To maintain comparability with the HCS sample and to address the aims of the current research, only participants aged 55 years and over from the ARMHS cohort (N = 1273) were considered for inclusion in the current (*primary* and *sub*) analyses. For the purposes of the current study, only participants who provided complete information on life time diagnosis variables (depression/anxiety, stroke, heart attack/angina, diabetes, high cholesterol, hypertension) and adequate data on all other variables were included in the current analyses; that is, at least 75% of the item data used to construct primary analysis model variables (primary outcome and predictor variables) and item responses to the Kessler 10 (see *Missing data:* for handling of missing data). Of the N = 4732 participants in the combined sample, 92.2% (N = 4364) provided adequate data for inclusion in the current analyses (see Additional file 1: Figure S1 for further information).

### Measures

#### Dependent variables (primary and sub-analysis)

*HRQoL impairment.* Self-rated health outcomes were assessed using the Assessment of Quality of Life (AQoL-6D), a 20-item self-report measure of HRQoL and general functioning [46]. The AQoL-6D forms six domains characterised as ‘Independent living’, ‘Relationships’, ‘Mental health’, ‘Coping’, ‘Pain’, and ‘Senses’ [46]. These domain scores form two higher-order factors representing the psychological (Mental health and Coping subscales; range = 1.00-5.00) and physical (Independent living, Relationships, Pain, Senses; range = 1.00-4.88) aspects of HRQoL impairment, with higher scores indicating greater impairment [39]. Significant impairment is indicated by scores greater than 1 standard deviation (SD) above the mean; scores were standardized using normative means and SD values for the physical (mean = 1.73, SD = 0.45) and psychological (mean = 1.98, SD = 0.50) HRQoL domains, from our earlier paper [39].

#### Predictor variables

Unless otherwise stated, all predictor variables were used in both the primary and sub-analysis.

##### *Demographic variables.*

Age, gender, level of education, marital status and retirement.

##### *Cardiovascular and affective conditions.*

Both cohorts administered items regarding lifetime self-reported diagnoses, including cardiovascular conditions (heart attack/angina or stroke) and depression/anxiety (ARMHS: ‘Has a doctor *EVER* told you that you have…’; HCS: ‘Have you ever been diagnosed with…’).

##### *Other health related indicators.*

Self-reported diagnoses of metabolic health risk factors: diabetes, hypertension, and high cholesterol.

##### *Current smoking.*

A common yes/no index of current smoking behaviour was constructed from the HCS and ARMHS measures of smoking behaviour.

##### *Obesity.*

Height and weight measurements were undertaken as part of a battery of clinical measures recorded by the HCS, while the ARMHS obtained these measurements through self-reported survey responses. To address the potential for bias in self-reporting height and weight measurements, correction equations were used based on 2007–2008 Australian national survey data [47], which adjusts for known biases in self-reported height and weight by participant age and gender. Body Mass Index was calculated as weight in kilograms divided by height in metres squared and values ≥ 30 used to classify obesity.

##### *Social support.*

Both cohorts collected conceptually related social support measures at baseline and follow-up. A composite index of social support, representing the network (number of supporting friends and relatives, the frequency of contact with these individuals, and involvement in organised social groups) and personal (access to close personal relationships) features of social support has been constructed for the purposes of the xTEND project [48]. For standardization purposes, grand means and SDs for index components were used to create the composite index.

##### *Recent adverse life events (secondary analysis only).*

A self-report questionnaire was used to assess the number of adverse life events experienced in the last 12 months (range 0–12) (i.e. ‘has a member of your family died?’, ‘have arguments or marital difficulties with your partner worsened?’, ‘have you had a major financial crisis?’) [49].

##### *Perceived financial difficulty (secondary analyses only).*

Assessed using a similar single item question to that used by the Household, Income and Labour Dynamics in Australia Survey [50], namely ‘Given your current needs and financial responsibilities, would you say that you and your family are’ ‘prosperous’, ‘very comfortable’, ‘reasonably comfortable’, ‘just getting along’, ‘poor’, or ‘very poor’, with higher scores indicating a poorer financial position (range 1–6).

### Contextual factors

#### Remoteness (primary analysis only)

Participant remoteness was classified using the Accessibility/Remoteness Index of Australia Plus ARIA+: [51] and geocoded using participant’s postal code. The ARIA + is a continuous index ranging from 0.00-15.00 (higher scores indicating greater remoteness) that is calculated based on the size of the nearest service centre and its average estimated road distance from the location. For descriptive purposes, these scores were collapsed into four categories of remoteness: major cities (range = 0.00-0.20); inner regional areas (range = 0.21-2.40), outer regional areas (range = 2.41-5.92), and remote/very remote areas (range > 5.92) [51].

#### Social capital (sub-analysis only)

Social capital was assessed using the Sense of Community Index [52] among the ARMHS cohort. This index comprises a 12 item true/false self-report questionnaire assessing an individual’s psychological sense of belonging to a community, with higher scores indicating greater social capital (range 0–12).

#### District level social capital and health service characteristics (secondary analyses only)

Data from the 2006–2009 NSW Adult Population Health Surveys (NSW Population Health Surveys*,*http://www.health.nsw.gov.au/surveys/Pages/default.aspx*)* were obtained from the Centre for Epidemiology and Evidence, NSW Health and combined to examine the social capital and health service characteristics of four levels of community remoteness. Data from this period were selected to coincide with the collection of baseline data from the HCS (2004–2007) and ARMHS (2007–2009). The NSW Adult Population Health Survey is an annual telephone survey of approximately 12,000 people aged 16 and above who are randomly selected from all area health services across NSW. The survey is conducted between February and December each year [53] and achieved response rates of 59-64% between 2006 and 2009. Data were weighted in accordance with procedures adopted by the NSW Adult Population Health Survey [53] to adjust for differential non-response rates by gender, age and by population estimates for each health service area, with raw sample sizes for surveyed remote/very remote participants maintained. *Social capital* was measured using nine items described by Onyx and Bullen [7] as best reflecting components of social capital (i.e. ‘participating in the local community’, ‘feelings of trust and safety’ and ‘neighbourhood connections’). Responses were provided on a four point Likert scale, with higher ratings indicating higher social capital. We constructed a mean total social capital score based on a minimum of six items to maximise the number of items and observations retained while preserving scale associations with related variables. An index of *Health service accessibility* was constructed using responses to the question ‘Do you have any difficulties getting health care when you need it?’. Persons who reported any need for health care (97.3%) provided either a ‘yes’ or ‘no’ response and were included in the current analyses.

### Data analysis

Analyses were conducted using SPSS (v.20; IBM Corporation, Armonk NY, USA) and graphs produced using SPSS and Microsoft Excel 2010. For an account of data utilised from different datasets for purposes of *primary*, *secondary* and *sub*-*analyses*, see Additional file 1: Figure S1. Continuous variables were described using means and SDs and categorical variables using frequencies and percentages. Effect sizes for group comparisons were expressed as the proportion of sample variance explained: eta-squared for ANOVA and Cramer’s V for chi-square tests. Effects were considered consistent if their direction and significance as predictors of HRQoL impairment were replicated across all statistical models. Some alternative versions of the regression models reported here are presented in Additional file 1: (Tables S1 to S3), together with simple correlations with the outcome measures.

#### Missing data

To address potential bias caused by the exclusion of persons with missing data in ARMHS and HCS studies, five datasets predicting these missing values were generated using the inbuilt SPSS multiple imputation procedure, following the recommendations of Graham [54]. Items from the Kessler 10 [55] were included in the multiple imputation procedure as potentially important model variables along with all predictor and dependent model variables in the primary analyses. Overall, 1% of data were imputed. Body mass index was by far the most frequently missing information (9.5% missing data) however no differences in obesity as a predictor were observed for models using imputed and non-imputed data. Pooled estimates are reported for all descriptive and inferential statistics.

#### Primary analyses

Dependent variables displayed approximate normal distributions, though physical HRQoL impairment displayed a slight positive skew. Two sets of multivariate linear regressions were used to identify factors associated with physical and psychological HRQoL impairment. Overall model fit and change in model fit by step were assessed using the R^2^ statistic. To assess whether the HRQoL impairment associated with a lifetime diagnosis of cardiovascular or affective conditions was influenced by remoteness, dichotomous indicators of whether the participant reported life time diagnoses of a cardiovascular (stroke, heart attack/angina) or affective (depression/anxiety) condition were produced and interaction terms generated with remoteness (Z score) to model their two and three way interactions [cardiovascular by affective by (Z)remoteness]. To assess the influence of cohort membership, each regression was run twice: once with cohort included as the last step of the regression (examining its residual contribution) and once with cohort membership included in the first step of the regression (examining its aggregate contribution); this approach facilitates an assessment of cohort membership as a potential effect modifier, which may arise due to a number of factors (e.g., due to participant characteristics, or differences in wording of survey items). The remaining variables were entered into the main analysis model in six steps: demographic factors; life time cardiovascular and affective conditions; the additional explanatory value of other health related indicators; contextual factors; two-way interaction terms; three-way interaction terms. Where present, interactions were explored by plotting the association of the HRQoL factor with the probability of diagnosis by each level of the effect modifier. An α < .01 was used as a significance threshold, as a partial control for the number of statistical tests, with marginal effects (*p* < .05) also noted.

#### Secondary analyses

To examine whether remoteness was associated with resources theorized to influence health outcomes in NSW, secondary data from the NSW Population Health Survey were used to examine the associations of social capital and health service accessibility with four descriptive categories of remoteness. Community members aged 16 and over (N = 42155; mean age = 54.38, SD = 18.03; male gender = 39.3%) provided data to the NSW Adult Population Health Survey during 2006–2009, with 49.5% residing in major cities, 28.2% from inner regional, 19.4% from outer regional, and 2.9% from remote/very remote areas. The corresponding weighted values were: mean age = 45.10, SD = 18.10; male gender = 49.3%; with 66.0% residing in major cities, 22.1% from inner regional, 10.7% from outer regional, and 1.2% from remote/very remote areas.

#### ARMHS sub-analyses

To further examine the possible effects of health-relevant community resources on HRQoL impairment, we analysed data from the ARMHS cohort which included a direct assessment of social capital; this measure was substituted as the contextual variable in the analysis, in place of remoteness, in conjunction with two other known explanatory variables: 12 month adverse life events and perceived financial difficulty. Participants from the primary analysis who did not have complete data for the social capital, adverse life events and perceived financial difficulty measures were excluded from this analysis. To assess whether the HRQoL impairment associated with a lifetime diagnosis of cardiovascular or affective conditions was influenced by social capital, dichotomous indicators of cardiovascular or affective conditions were used to generate interaction terms with social capital (Z score) to model their two and three way interactions [cardiovascular by affective by (Z)social capital]. Additionally, to assess whether the HRQoL impairment associated with perceived financial difficulty was influenced by social capital, the interaction of these variables was produced and entered with the other two-way interactions in the model. To facilitate comparison of this extended predictor model (including financial difficulty and adverse life events) with the model examined in the primary analysis, a model without the additional predictor variables was examined and is provided in Additional file 1: Table S3.

## Results

### Primary analysis: the influence of remoteness on HRQoL impairment associated with cardiovascular and affective conditions from the xTEND study

For the N = 4364 participants aged 55 and over who provided adequate data for inclusion in the current analyses overall descriptive statistics are reported in Table 1 and are compared by cohort membership. The mean age of participants was 66 years and approximately half were female. There were few differences between HCS and ARMHS participants, although a higher proportion of HCS participants had completed 12 or more years of formal education and a higher proportion of ARMHS participants were current smokers, trends consistent with those of rural populations in Australia [3]. As indices of social support were standardized within each cohort to facilitate assessment of this variable’s association with outcome variables across groups, no group comparisons were conducted. ARMHS participants reported significantly lower physical and psychological HRQoL impairment, with the latter representing a more marked difference (0.17 vs. 0.41 standardised units).

**Table 1:** Descriptive statistics and comparison of HCS (N = 3118) and ARMHS (N = 1246) participants

	**HCS**	**ARMHS**	***p***	**Overall**
**Demographic factors *%***				
Age *Mean (SD)*	66.17 (7.78)	66.10 (7.68)	.783	66.15 (7.75)
Female	52.92	56.82	.020	54.03
12 + years education	76.77	60.63	<.001	72.16
Married/de facto	72.95	71.04	.209	72.41
Retired	62.69	61.04	.314	62.22
**Cardiovascular &affective conditions *%***				
Stroke	4.14	4.57	.508	4.26
Heart attack	12.12	11.64	.680	11.98
Any CVD	15.14	15.09	.999	15.12
Depression/anxiety	20.97	23.84	.042	21.79
CVD & depression	3.59	3.85	.369	3.67
**Other health related indicators *%***				
Diabetes	11.10	11.96	.428	11.34
Obese	34.96	31.65	.041	34.02
Current smoker	7.66	10.13	.009	8.36
High cholesterol	39.58	38.84	.681	39.37
Hypertension	47.11	47.03	.973	47.09
Social support *Mean (SD)*	0.01 (0.81)	0.01 (0.83)	.	0.01 (0.82)
**Quality of life impairment***Mean (SD)*				
Physical	1.82 (0.47)	1.75 (0.47)	<.001	1.80 (0.47)
*standardized*	*0.20 (1.05)*	*0.03 (1.03)*		*0.16 (1.05)*
Psychological	2.04 (0.51)	1.84 (0.52)	<.001	1.99 (0.52)
*standardized*	*0.13 (1.01)*	*−0.28 (1.05)*		*0.01 (1.04)*
**Contextual factors***Mean (SD)*				
Remoteness	0.05 (0.12)	3.96 (3.08)	<.001	1.16 (2.41)

Note: Reported statistics are based on pooled results (across multiple imputation datasets); *CVD* cardiovascular disease, *ARMHS* Australian Rural and Remote Mental Health Study, *HCS* Hunter Community Study; quality of life impairment scores were standardized using normative means and standard deviations for the physical and psychological HRQoL domains reported in Allen et al. [39].

#### Physical HRQoL impairment

The left hand columns of Table 2 show the results of the regression model predicting physical impairment. Demographic indices, cardiovascular and affective conditions and other health indicators (Steps 1 to 3) contributed approximately 23.8% of the 24.6% variation explained by the model. There were no two- or three-way interaction effects of cardiovascular, affective conditions and remoteness on physical impairment (Steps 5 and 6). All demographic, health condition and related indicators, with the exception of high cholesterol, were significantly associated with physical impairment and the magnitude and significance of these predictors was largely unaffected by cohort membership (see Additional file 1: Table S1 for comparisons with cohort membership modelled in the first regression step and Figure S2 for the influence of cohort on the association of remoteness with physical HRQoL).

**Table 2:** Primary analysis: hierarchical linear regression analysis of the correlates of physical and psychological quality of life impairment (N = 4364)

		**Physical impairment**				**Psychological impairment**			
		**β**	***p***	**Step R**^**2**^	***p***	**β**	***p***	**Step R**^**2**^	***p***
Step 1	**Demographic factors**			0.067	<.001			0.013	<.001
	(Z)Age	0.16	<.001			−0.04	.014		
	Female	−0.05	.002			0.07	<.001		
	12 + years education	−0.10	<.001			−0.02	.116		
	Married/de facto	−0.08	<.001			−0.07	<.001		
	Retired	0.06	<.001			0.01	.500		
Step 2	**Cardiovascular &affective conditions**			0.069	<.001			0.114	<.001
	Stroke	0.10	<.001			0.07	<.001		
	Heart-attack/angina	0.14	<.001			0.06	<.001		
	Depression/anxiety	0.19	<.001			0.32	<.001		
Step 3	**Other health related indicators**			0.102	<.001			0.120	<.001
	Diabetes	0.09	<.001			0.01	.322		
	Obesity	0.18	<.001			0.07	<.001		
	Current smoker	0.04	.006			0.03	.029		
	High cholesterol	−0.01	.674			0.00	.999		
	Hypertension	0.04	.003			0.02	.153		
	Social support	−0.22	<.001			−0.34	<.001		
Step 4	**Contextual factors**			0.003	<.001			0.018	<.001
	(Z)Remoteness	−0.06	<.001			−0.14	<.001		
Step 5	**Interactions (2-way)**			0.001	.353			0.002	.007
	Cardiovascular*(Z)Remoteness	0.01	.593			0.02	.103		
	Cardiovascular*Depression	0.01	.387			0.04	.010		
	Depression*(Z)Remoteness	−0.02	.126			−0.03	.074		
Step 6	**Interactions (3-way)**			0.000	.437			0.000	.950
	Cardiovascular*Depression* (Z)Remoteness	−0.01	.438			0.00	.966		
Step 7	**Cohort**	0.10	<.001	0.004	<.001	0.20	<.001	0.018	<.001
**Model diagnostics**									
Significance (F value)		70.85, p < .001				86.64, p < .001			
*R*^*2*^		24.6%				28.5%			
Adjusted *R*^*2*^		24.3%				28.2%			

Note: Reported statistics are based on pooled results (across multiple imputation datasets).

There was a small but statistically significant effect of remoteness on physical impairment (accounting for under one percent of the explained variation), which disappeared when cohort membership was accounted for (see Additional file 1: Table S1, β = 0.02, *p* = 0.404). Since the cohorts were chosen primarily because they differed in remoteness (i.e., it is a group defining characteristic, see Table 1), this finding suggests that continuous scores on the remoteness index added little to prediction beyond the urban versus rural comparison. On the other hand, cohort effects remained (see Table 2, Step 7) even after all other factors (including remoteness) were controlled for, suggesting that other (non-assessed) cohort related factors were also associated with impaired physical HRQoL; however, in both of these models cohort accounted for under one percent of the explained variation (see Additional file 1: Table S1).

#### Psychological HRQoL impairment

The right hand columns of Table 2 show the results for the regression model predicting psychological impairment. Demographic indices, cardiovascular and affective conditions and other health indicators (Steps 1 to 3) contributed approximately 24.7% of the 28.5% variation explained by the model. All steps significantly added to the model, with the exception of the three-way interaction (Step 6). Several factors were significantly associated with psychological impairment (most notably, a lifetime affective condition and lower social support) and the magnitude and significance of these predictors were largely unaffected by the inclusion of cohort membership in the first step of the model (see Additional file 1: Table S2).

However, the two factors that were associated with cohort differences in Table 1 (education and current smoking status) only reached the threshold for statistical significance when cohort membership was controlled. That is, after controlling for urban versus rural differences, lower education and smoking were associated with higher psychological impairment. Conversely, remoteness was significantly associated with lower psychological impairment (accounting for 1.8% of the explained variation, see Table 2) but not after cohort membership was controlled (see Additional file 1: Table S2 and Figure S2), raising doubt about the value of continuous scores on the remoteness index beyond the urban versus rural comparison. In addition, the effects of a lifetime affective condition on psychological impairment were worse for those who also reported a lifetime cardiovascular condition (Step 5, *p* < .010), which remained after controlling for cohort (see Additional file 1: Table S2; β = 0.04, *p* = 0.014).

### Secondary analyses: contextual correlates of remoteness from the NSW Adult Population Health Survey

Descriptive statistics for social capital and health service accessibility by remoteness category are displayed in Table 3. An overall influence of remoteness category on social capital of small effect size was observed (*F*(3, 74909) = 1042.53, *p* < .001, eta-squared = .040). Post hoc tests indicated that all group differences were statistically significant (*p* < .001) with the exception of outer regional versus remote/very remote areas (*p* = .408). Residents of major cities reported the lowest social capital, followed by inner regional, outer regional and remote-very remote areas. Similarly, a chi-squared test indicated that the proportion of people with difficulties accessing health care varied by remoteness category, with a medium effect size (*X*^*2*^(3) = 3455.09, *p* < .001, Cramer’s *V* = .191). Post hoc tests indicated that all group differences were statistically significant (*p* < .001) with the exception of outer regional versus remote-very remote areas (*p* = .995). Major cities had the highest proportion of persons reporting no difficulty accessing health care when needed, followed by inner regional areas and outer regional and remote/very remote areas. Overall 1.79% of participants reported that they did not need health services and the proportion of these participants did not vary by remoteness category (*X*^*2*^(3) = 0.42, *p* = .936). These secondary analyses support an association between remoteness and both social capital and health service accessibility, though findings of no difference between outer-regional and remote-very remote areas also suggest a level of insensitivity of our remoteness index to these underlying community characteristics.

**Table 3:** Secondary analysis of the subjective social capital and health service accessibility by remoteness category from NSW adult population health surveys (years: 2006–2009)

		**Major City**	**Inner Regional**	**Outer Regional**	**Remote/Very Remote**	**Statistically significant comparisons**
		**(MC)**	**(IR)**	**(OR)**	**(R/VR)**	
Social capital	N	49,267	16,712	8030	902	MC < IR < OR & R/VR
	Mean	2.44	2.62	2.73	2.70	
	SD	0.52	0.53	0.52	0.53	
Health service accessibility	N	62,495	20,938	10,134	1126	MC > IR > OR & R/VR
	%	88.3	77.6	68.4	68.4	

Note: Comparisons based on one-way ANOVA with Scheffé post hoc tests (social capital) or chi-squared tests (health service accessibility); significant follow-up comparisons: all *p* < .001; Health service accessibility % indicates the proportion of persons reporting no difficulty accessing services when needed; results are weighted to adjust for differential non-response rates by gender, age, remoteness and by population estimates for each health service area.

### Sub-analyses: the influence of social capital on HRQoL impairment associated with cardiovascular, affective conditions and perceived financial difficulty from the ARMHS study

Those included in the ARHMS sub-analysis (N = 1176) were marginally younger (mean = 65.96, SD = 7.54 vs. N = 70, mean = 68.34, SD = 9.50; *p* = .012), had experienced fewer adverse life events (mean = 1.32, SD = 1.37 vs. N = 51, mean = 1.84, SD = 1.92; *p* = .009) and reported less perceived financial difficulty (mean = 3.18, SD = 0.76 vs. N = 54, mean = 3.48, SD = 0.92; *p* = .008) compared to those excluded (N = 70). One-way ANOVA revealed a small significant effect of remoteness category on social capital ratings (*F*(2, 1202)= 6.93, *p* < .001, eta-squared = .011) with Scheffé post hoc tests indicating that participants from inner regional areas (N = 484, mean = 9.06, SD = 2.13) reported significantly lower (*p* < .001) social capital than those from remote/very remote areas (N = 285, mean = 9.65, SD = 1.99). Outer regional participants did not differ from those of the other areas (N = 434, mean = 9.14, SD = 2.37).

Two models were used to assess the influence of social capital on physical and psychological impairment: 1) a model identical to that used in the primary analysis but with contextual factors represented by an individual’s rating of social capital; and 2) an expanded model including the influence of recent adverse life events and perceived financial difficulty. As individual level ratings of social support and social capital are likely to be correlated, social capital was included at a subsequent step in the regression model to enhance our capacity to examine the benefits of community level support above those attributable to an individual’s propensity for close social relationships. Results of the former analyses are presented in Additional file 1: Table S3 and the latter analysis in Table 4.

**Table 4:** Sub-analysis: hierarchical linear regression analysis of the correlates of physical and psychological quality of life impairment (N = 1176)

		**Physical impairment**				**Psychological impairment**			
		**β**	***p***	**Step R**^**2**^	***p***	**β**	***p***	**Step R**^**2**^	***p***
Step 1	**Demographic factors**			0.085	<.001			0.033	<.001
	(Z)Age	0.14	<.001			−0.14	<.001		
	Female	−0.08	.004			0.04	.157		
	12+ years education	−0.10	.002			−0.03	.333		
	Married/de facto	−0.08	.004			−0.11	<.001		
	Retired	0.14	<.001			0.12	<.001		
Step 2	**Cardiovascular &affective conditions**			0.080	<.001			0.122	<.001
	Stroke	0.08	.005			0.09	.001		
	Heart-attack/angina	0.16	<.001			0.08	.006		
	Depression/anxiety	0.22	<.001			0.33	<.001		
Step 3	**Other health related indicators**			0.133	<.001			0.129	<.001
	Diabetes	0.06	.029			0.02	.433		
	Obesity	0.12	<.001			0.04	.172		
	Current smoker	0.03	.266			0.03	.284		
	High cholesterol	−0.05	.043			−0.05	.041		
	Hypertension	0.07	0.01			0.04	.186		
	Social support	−0.16	<.001			−0.24	<.001		
	(Z)Adverse life events	0.13	<.001			0.14	<.001		
	(Z)Fin difficulty	0.22	<.001			0.19	<.001		
Step 4	**Contextual factors**			0.000	0.603			0.003	0.028
	(Z)Social capital	−0.01	.604			−0.06	.028		
Step 5	**Interactions (2-way)**			0.002	0.462			0.008	0.008
	(Z)Fin difficulty*(Z)Social capital	−0.04	.112			−0.07	.014		
	Cardiovascular*(Z)Social capital	0.02	.454			0.02	.480		
	Cardiovascular*Depression	0.02	.550			0.06	.053		
	(Z)Social capital*Depression	0.03	.285			−0.05	.145		
Step 6	**Interaction (3-way)**			0.001	0.329			0.00	0.790
	Cardiovascular*Depression*(Z)Social capital	−0.03	.329			−0.01	.790		
**Model diagnostics**									
Significance (F-value)		22.49, p < .001				22.01, p < .001			
*R*^*2*^		30.03%				29.58%			
Adjusted *R*^*2*^		28.69%				28.24%			

Note: Reported statistics are based on pooled results (across multiple imputation datasets). Fin difficulty = perceived financial difficulty.

**Figure 1:**
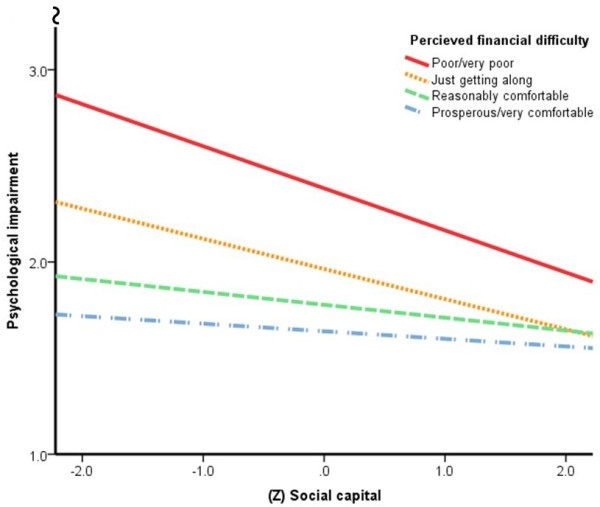
**Sub-analysis of the association of social capital and psychological impairment by perceived financial difficulty grouping.** Variability in psychological impairment attributable to social capital by perceived financial difficulty group**:** Poor-very poor (N = 37, R^2^ = .12); Just getting along (N = 337, R^2^ = .09); Reasonably comfortable (N = 615, R^2^ = .02); Prosperous/very comfortable (N = 781, R^2^ = .01). Overall variability in psychological impairment (main effects) attributable to perceived financial difficulty (R^2^ = .09) and social capital (R^2^ = .04).

The inclusion of 12 month adverse events and perceived financial difficulty (at Step 3) did not generally influence the direction or significance of model variables. However, a trend indicating that the impact of depression/anxiety on psychological impairment decreased with greater social capital (Table S3: *p* = .012) was no longer significant in the extended model. Instead, a trend indicating that the impact of financial difficulty on psychological impairment decreased with greater social capital (Table 4: *p* = .014) was observed in the extended model. The null result regarding the interaction of depression/anxiety and social capital in the extended model may be due to variance shared by financial difficulty and depression/anxiety and their interaction with social capital in the prediction of psychological impairment. Figure 1 illustrates this association by displaying univariate regression lines for ratings of social capital on psychological impairment for four financial difficulty subgroups. This figure indicates that increased social capital had the greatest positive psychological impact on persons experiencing financial difficulty, with little to no psychological impact on persons who perceived themselves as prosperous or very comfortable financially. There were no significant two- or three-way interactions of cardiovascular, affective conditions or social capital in the prediction of physical impairment in the extended model.

Results of the rural sub-analysis were consistent with those of the overall primary analysis reported in Table 2, although the effects of smoking on physical and psychological impairment were no longer apparent, nor was the effect of obesity on psychological impairment. However, retirement status was a predictor of psychological impairment and the influence of age on psychological impairment appeared to be greater. The associations of diabetes and hypertension with physical impairment were now of marginal significance though of similar magnitude. There was no association between social capital and physical impairment, though a marginal association with psychological impairment was observed, with increased social capital associated with decreased psychological impairment. Correlations reported in Additional file 1: Table S3 suggest that the simple association between social capital and psychological impairment was reduced in the multivariate model (−0.21 in Table S3 vs. -0.06 in Table 4), likely due to the inclusion of social support in the previous step.

## Discussion

This study reports population based findings about factors associated with HRQoL impairment in a sample of older persons from across urban-remote areas of NSW, as well as examining the impact of contextual factors and cardiovascular-affective condition comorbidity on these outcomes. Investigation of the moderating effect of cohort membership on these models revealed that, with the exception of factors directly associated with cohort membership (i.e. remoteness) there were few differences in the magnitude or significance of model predictors, supporting the validity of combining data across different cohorts. Current models explained approximately a quarter of the variance in physical and psychological impairment reported by participants, with demographic indices, cardiovascular and affective conditions and other health indicators accounting for most of the model variance. Physical impairment was consistently associated with increased age, male gender, lower education, being unmarried, retirement, a lifetime history of stroke, heart attack/angina, depression/anxiety, diabetes and hypertension, as well as current obesity and low social support. Psychological impairment was consistently associated with lower age, being unmarried, a lifetime history of stroke, heart attack/angina and depression/anxiety, as well as low social support. In the primary analyses, the influence of participant remoteness on HRQoL impairment was relatively small and varied with the model being examined. Remoteness tended to be more strongly associated with lower psychological impairment, reflecting overall urban versus rural differences, rather than more subtle changes in remoteness.

Previous research in the Australian context has also observed a rural advantage for psychological [56,57], but not physical quality of life [56] compared to urban participants. In line with previous research regarding the impact of comorbid physical and mental health conditions [26], trends suggesting that the lifetime occurrence of *both* affective and cardiovascular conditions was associated with greater psychological impairment than was explained by either diagnosis alone (*p* = .010) were observed, although they were not significant in the rural sub-analysis (*p* = .053). Moreover, there was no evidence that the impact of cardiovascular and affective conditions were influenced by remoteness. However, these findings must be interpreted with caution. Several factors suggest that chronic conditions may be more likely to be miss-classified or of greater severity in our rural populations, particularly: the decreased probability of receiving a diagnosis in remote areas where health services are less accessible [58,59]; the potential for increased severity at diagnosis; the relative infrequency with which rural populations with health conditions consult their physician; the reduced likelihood of surviving an acute health event; and evidence of migration of persons to less remote areas following diagnosis of mental health conditions [60]. Such biases in diagnostic classification (i.e. more persons incorrectly classified as not having the condition) in regional-remote areas would mean that the influence of disease on quality of life would be underestimated in these areas, though it would be difficult to determine the degree to which such an effect could be offset by the increased severity of diagnosed cases. Further, investigations regarding impact of migration patterns on mental health outcomes suggest migration from rural to urban areas to be associated with increased probability of depression, with decreased contact with friends and neighbours a particular burden in this group [61]. Thus, current results may be best characterised as representing the influence of current community remoteness on persons who have received these diagnoses.

Analyses of NSW Adult Population Health Survey data confirm observations that social capital increases [7] and experiences of health service accessibility decrease [59] with remoteness, though no differences were observed for either factor between outer regional and remote/very remote groups. Approximately 32% of persons in outer regional and remote/very remote areas reported difficulty accessing health services when needed compared to 12% living in major cities. Conversely, outer regional and remote/very remote participants reported levels of social capital approximately half a SD greater than their major city counterparts. These findings provide some support for the use of remoteness indices as a proxy for health related community characteristics, although they also tend to suggest that a three category classification would be sufficient (i.e., major city, inner regional, and other areas), and that the current remoteness indices lack greater sensitivity.

The impact of these supposedly opposing forces (increased social capital and decreased health service accessibility) upon health outcomes requires further research, though it is possible that in light of their co-variation, the protective effects of social capital reported here are under-estimated. For example, while we have observed no effect of social capital on physical HRQoL outcomes in our ARMHS sub-analyses, it may be that these community effects are offset by poorer health service accessibility. However, current results are in line with previous investigations of the influence of social capital on HRQoL in Australia, with social capital displaying a particular influence of social capital on psychological HRQoL [62]. Further, while the association of social capital with psychological HRQoL has been observed for both urban and rural participants, evidence suggests that social capital is associated with physical HRQoL only in urban populations [56]. This is consistent with the current null finding regarding the relationship of social capital with HRQoL in our rural sample and may be due to limitations on the capacity of social capital to influence physical health related behaviours in rural areas where health resources are limited.

As discussed above, in line with previous research our sub-analysis of ARMHS data revealed a marginal association of social capital with decreased psychological impairment when controlling for individual level variables such as social support. In our replication of the primary analysis (Additional file 1: Table S3), social capital influenced the association of affective conditions with psychological impairment; as social capital increased, persons with a lifetime diagnosis of depression/anxiety reported less psychological impairment. These effects were observed in the replication despite the fact that other major drivers of wellbeing were included in the model, such as personal social support. This effect was not significant in the extended model which included recent adverse life events, perceived financial difficulty and a marginal interaction of financial difficulty with social capital, suggesting that these variables shared a portion of the variance in psychological impairment accounted for by the social capital and affective disorder interaction. Both marginal interactions observed suggest that interrelated psychological burdens, such as affective disorders and financial difficulties, are similarly ameliorated by social capital. The previously observed trend for comorbid lifetime diagnoses of cardiovascular and affective disorder to be associated with psychological impairment was of similar magnitude but not significant in this subsample (*p* = .053). The ARMHS cohort sub-analysis also confirmed the influence of recent adverse life events and perceived financial difficulty on HRQoL impairment. Evidence for a moderating effect of social capital on the negative effect of financial difficulty on psychological HRQoL impairment was also observed.

Comparisons between the corresponding analyses (Table 4 vs. Additional file 1: Table S3) show an increment in explained variation of approximately six percent with the inclusion of the additional predictors (adverse life events, perceived financial difficulty and the interaction of financial difficulty and social capital). We acknowledge that the individual level measures of social capital used in these analyses may themselves be influenced by each person’s own psychological HRQoL. However, the patterns of social capital in this sample are consistent with those observed in the NSW data and elsewhere [5-7], namely, increased social capital across rural locations, suggesting this is a potentially health-sustaining quality of rural living, particularly for those with a history of affective conditions. These results are consistent with previously hypothesised and observed ameliorating influences of social capital on stressful situations and events [5]. It is possible that community engagement and support plays a greater role in supporting psychological wellbeing of persons with financial difficulties, suggesting that they have greater engagement with the community in maintaining their psychological wellbeing. Given that these variables were only assessed in the rural-remote ARMHS cohort and not the overall xTEND sample, a limitation of these analyses is that they do not include persons from urban areas and thus the effects and interactions reported here are likely be truncated representations of the effects present in the community at large.

Current findings have practical implications for research into the influence of comorbidity and context on health outcomes, particularly in Australia. This report informs concerns raised by the 2012 National Report Card on Mental Health and Suicide Prevention regarding the physical health of persons affected by mental illness [30], particularly in light of the burden of cardiovascular disease in these populations. Current results build on past observations of an effect of physical-mental comorbidity on increased days out of role and high health service usage [26], short term disability and suicidal ideation [63], decreased HRQoL [28] and general disability beyond that of diagnoses in isolation [29]. Our results tend to suggest that the disability associated with comorbidity may have a stronger association with psychological HRQoL. In any event, all of the analyses demonstrated clear independent linkages between lifetime cardiovascular and affective conditions and current physical and psychological HRQoL impairment (accounting for between 6.9% and 12.3% of the explained variation).

The strengths of this study are its consideration of data from large community based samples and access to a depth of health information from participants across the spectrum of urban-remote communities that is unprecedented in Australia. Our models include a range of bio-psychosocial risk factors that are not only potentially important for understanding the relationship of physical and mental disorders with HRQoL but which also enable us to tease out some of the contextual, rather than behavioural, influences of remoteness on HRQoL outcomes (such as increased rates of smoking). It should be noted that response rates for these surveys were relatively low, particularly for the oldest persons contacted, among whom the impact of disease on participation is likely to be high. Therefore, we infer that the current subset of participants represents a relatively healthy sub-sample of the population at large, and that the impacts of disease on quality of life depicted here are potentially weaker than those which would be observed in the general population.

The study has several other limitations. Firstly, the use of self-reported lifetime diagnoses for health conditions meant that these variables may reflect a range of symptoms that may not be current and do not account for duration or severity. In the current analyses, self-reported life time diagnoses of affective conditions were among the strongest predictors of both physical and psychological impairment. However, the impact of lifetime health conditions may be variable and the effects of current or recent experiences of these conditions on HRQoL impairment may be greater than those represented here. Secondly, apart from the obvious urban versus rural difference, it is unclear what other cohort related factors may have contributed to differences in mean HRQoL impairment. Finally, it should be noted that our urban population was drawn from a major regional industrial city and thus the current observations of factors influencing HRQoL may not generalise to other urban contexts. In particular, differences between characteristics of major urban locations, which are not necessarily delineated by population density or distance from services, and populations residing within these areas, may impact the experiences of social capital and health service accessibility and their association with health between urban centres [13].

A strength of the current study is that our primary outcome measure, the AQoL-6D, has been shown to display metric invariance across these cohorts [39], suggesting that the same constructs are tapped by this measure in both groups. Further, the inclusion of cohort membership in the models did not substantially change the significance or magnitude of model variables as predictors of HRQoL outcomes. Some aspects of the greater impairment reported by the HCS cohort may reflect cohort differences not assessed by our current measures. For example, a component of the observed differences may be a result of the HCS’s focus on recruitment of older persons with an interest in feedback about their health and by the ARMHS protocol of screening out participants with poor hearing and cognitive performance. However, these potential influences are likely to be small. Equally, the residual cohort effects observed in Table 2 (Step 7) may still be due to important elements of urban versus rural differences, but which are simply aspects not captured by the existing remoteness indices. Thirdly, the cross-sectional design of the current study means that we cannot assume that the HRQoL impairments observed here were actually caused by the variables under investigation.

### Conclusion

The findings from this study support the influence of social capital on HRQoL impairment, with particular focus on co-existing affective and cardiovascular conditions, two of the most common causes of disease burden in the Australian community. Findings suggest that the psychological impairment experienced by persons affected by lifetime affective conditions may be influenced by comorbid cardiovascular conditions (and vice versa) and by low social capital. Awareness of the compounded effects of physical-mental comorbidity on psychological impairment in these populations is necessary to equitably address their experiences of health conditions. Greater remoteness was associated with higher levels of social capital, reflected in overall urban/rural differences in psychological impairment. The findings suggest that personal social capital may ameliorate the psychological impairment associated with affective disorders and financial difficulties. Initiatives with a focus on social support and social engagement may make help to improve the HRQoL of older persons in the Australian community.

## Abbreviations

AQoL-6D: Assessment of Quality of Life-6D; ARMHS: Australian Rural Mental Health Study; HCS: Hunter Community Study; xTEND: eXtending Treatments, Education and Networks for Depression; CVD: Cardiovascular disease; ARIA+: Accessibility/Remoteness Index of Australia plus.

## Competing interests

The authors declare that they have no competing interests.

## Authors’ contributions

BJK and TJL led the ARMHS study and JRA led the HCS study from 2010. BJK, KJI, JRA and TJL led the program of research associated with the combination of these studies as the eXtending Treatments, Education and Networks for Depression (xTEND) project. JA and TJL undertook the statistical modelling and generated the results. All authors provided interpretation of the results. JA drafted the manuscript and all authors contributed to its editing. All authors read and approved the final manuscript.

## Supplementary Material

Additional file 1: Figure S1 Flow chart depicting inclusion of participants from data sources and N included in each analysis; # subset of Australian Rural Mental Health Study participants aged 55 and over; NSW, New South Wales; NSC, raw N for the analysis of social capital by remoteness; NHSA, raw N included in the analysis of health service accessibility by remoteness; NSCw, weighted social capital N; NHSAw weighted health service accessibility N. **Table S1.** Primary analysis: Hierarchical linear regression analysis of the correlates of physical quality of life impairment with (Model 2) and without (Model 1) cohort included in the first step of the regression model (N = 4364). **Table S2.** Primary analysis: Hierarchical linear regression analysis of the correlates of psychological quality of life impairment with (Model 2) and without (Model 1) cohort included in the first step of the regression model (N = 4364). **Figure S2.** Standardized mean physical and psychological quality of life impairment scores by cohort and remoteness category. Note: HCS, Hunter Community Study; ARMHS, Australian Rural Mental Health Study. **Table S3.** Sub-analysis: Hierarchical linear regression analysis of the correlates of physical and psychological quality of life impairment (replication of the primary analyses) – ARMHS sub analysis (N = 1176). Click here for file
