# Supplementary material for: Quality of life impact of cardiovascular and affective conditions among older residents from urban and rural communities
Source: Health Qual Life Outcomes. 2013 Aug 14;11:140. doi: 10.1186/1477-7525-11-140 (PMC3751480; doi:10.1186/1477-7525-11-140)
Supplement: Additional file 1: Figure S1 — Flow chart depicting inclusion of participants from data sources and N included in each analysis; # subset of Australian Rural Mental Health Study participants aged 55 and over; NSW, New South Wales; NSC, raw N for the analysis of social capital by remoteness; NHSA, raw N included in the analysis of health service accessibility by remoteness; NSCw, weighted social capital N; NHSAw weighted health service accessibility N. Table S1. Primary analysis: Hierarchical linear regression analysis of the correlates of physical quality of life impairment with (Model 2) and without (Model 1) cohort included in the first step of the regression model (N = 4364). Table S2. Primary analysis: Hierarchical linear regression analysis of the correlates of psychological quality of life impairment with (Model 2) and without (Model 1) cohort included in the first step of the regression model (N = 4364). Figure S2. Standardized mean physical and psychological quality of life impairment scores by cohort and remoteness category. Note: HCS, Hunter Community Study; ARMHS, Australian Rural Mental Health Study. Table S3. Sub-analysis: Hierarchical linear regression analysis of the correlates of physical and psychological quality of life impairment (replication of the primary analyses) – ARMHS sub analysis (N = 1176). [file 1477-7525-11-140-S1.pdf]

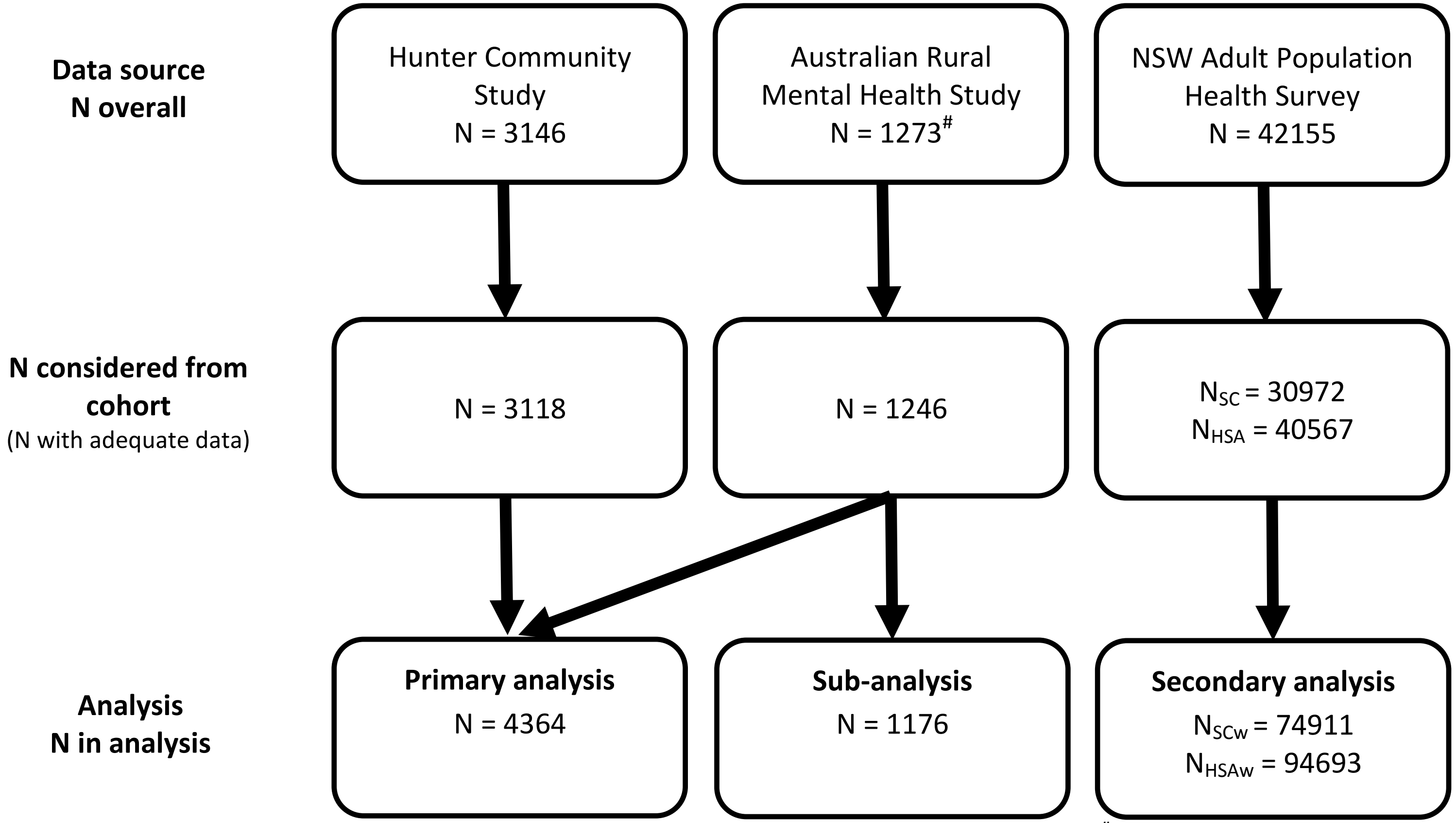

Figure S1. Flow chart depicting inclusion of participants from data sources and N included in each analysis; # subset of Australian Rural Mental Health Study participants aged 55 and over; NSW, New South Wales; N_{SC}, raw N for the analysis of social capital by remoteness; N_{HSA}, raw N included in the analysis of health service accessibility by remoteness; N_{SCw}, weighted social capital N; N_{HSAw} weighted health service accessibility N.

Table S1. Primary analysis: Hierarchical linear regression analysis of the correlates of physical quality of life impairment with (Model 2) and without (Model 1) cohort included in the first step of the regression model (N = 4364).

		Model 1				*Model 2*			
	r	β	*p*	Step R^2	*p*	β	*p*	Step R^2	*p*
**Step: Cohort**		.	.	.	.	0.07	<.001	0.005	<.001
**Step: Demographic factors**				0.067	<.001			0.069	<.001
(Z)Age	0.22	0.16	<.001			0.16	<.001		
Female	-0.02	-0.05	.002			-0.04	.003		
12+ years education	-0.12	-0.10	<.001			-0.11	<.001		
Married/de facto	-0.11	-0.08	<.001			-0.08	<.001		
Retired	0.15	0.06	<.001			0.06	<.001		
**Step: Cardiovascular & affective conditions**				0.069	<.001			0.070	<.001
Stroke	0.14	0.10	<.001			0.10	<.001		
Heart-attack/angina	0.20	0.14	<.001			0.14	<.001		
Depression/anxiety	0.19	0.19	<.001			0.20	<.001		
**Step: Other health indicators**				0.102	<.001			0.100	<.001
Diabetes	0.16	0.09	<.001			0.09	<.001		
Obesity	0.22	0.18	<.001			0.18	<.001		
Current smoker	0.05	0.04	.006			0.04	.003		
High cholesterol	0.07	-0.01	.674			-0.01	.624		
Hypertension	0.16	0.04	.003			0.04	.003		
Social support	-0.25	-0.22	<.001			-0.22	<.001		
**Step: Contextual factors**				0.003	<.001			0.000	.404
(Z)Remoteness	-0.04	-0.06	<.001			0.02	.404		
**Step: Interactions (2-way)**				0.001	.353			0.000	.513
Cardiovascular*(Z)Remoteness	0.01	0.01	.593			0.01	.718		
Cardiovascular*Depression	0.01	0.01	.387			0.01	.429		
Depression*(Z)Remoteness	-0.02	-0.02	.126			-0.02	.203		
**Step: Interactions (3-way)**				0.000	.437			0.000	.435
Cardiovascular*Depression*(Z)Remoteness	-0.02	-0.01	.438			-0.01	.436		
**Step: Cohort**	0.07	0.10	<.001	0.004	<.001	.	.	.	.
**Model diagnostics**									
Significance (F-value)					70.85, p < .001				
R^2					24.6%				
Adjusted *R^2*					24.3%				

Note: Reported statistics are based on pooled results. With the exception of those for the remoteness predictor, beta coefficients displayed a high level of stability across models. The significant effect of cohort in predicting quality of life impairment once remoteness is accounted for (Model 1) suggests other (non-assessed) factors associated with cohort membership were also associated with quality of life outcomes. The univariate associations of indicators with physical HRQoL impairment are also presented using Pearson's correlation coefficients (r); with the possible exceptions of retirement and life time diagnosis of hypertension, these correlations were largely consistent with the multivariate associations, suggesting that model design had limited influence on the reported effects.

Table S2. Primary analysis: Hierarchical linear regression analysis of the correlates of psychological quality of life impairment with (Model 2) and without (Model 1) cohort included in the first step of the regression model (N = 4364).

		Model 1				*Model 2*			
	r	β	*p*	Step R^2	*p*	β	*p*	Step R^2	*p*
**Step: Cohort**		.	.	.	.	0.18	<.001	0.031	<.001
**Step: Demographic factors**				0.013	<.001			0.016	<.001
(Z)Age	-0.02	-0.04	.014			-0.05	.006		
Female	0.08	0.07	<.001			0.07	<.001		
12+ years education	-0.03	-0.02	.116			-0.06	<.001		
Married/de facto	-0.08	-0.07	<.001			-0.07	<.001		
Retired	0.01	0.01	.500			0.01	.616		
**Step: Cardiovascular & affective conditions**				0.114	<.001			0.117	<.001
Stroke	0.08	0.07	<.001			0.08	<.001		
Heart-attack/angina	0.06	0.06	<.001			0.06	<.001		
Depression/anxiety	0.34	0.32	<.001			0.33	<.001		
**Step: Other health indicators**				0.120	<.001			0.119	<.001
Diabetes	0.04	0.01	.322			0.02	.224		
Obesity	0.10	0.07	<.001			0.06	<.001		
Current smoker	0.08	0.03	.029			0.03	.009		
High cholesterol	0.04	0.00	.999			0.00	.879		
Hypertension	0.05	0.02	.153			0.02	.128		
Social support	-0.37	-0.34	<.001			-0.34	<.001		
**Step: Contextual factors**				0.018	<.001			0.000	.647
(Z)Remoteness	-0.12	-0.14	<.001			0.01	.647		
**Step: Interactions (2-way)**				0.002	.007			0.001	.029
Cardiovascular* (Z)Remoteness	0.02	0.02	.103			0.02	.197		
Cardiovascular*Depression	0.03	0.04	.010			0.04	.014		
Depression*(Z)Remoteness	-0.02	-0.03	.074			-0.02	.205		
**Step: Interactions (3-way)**				0.000	.950			0.000	.952
Cardiovascular*Depression*(Z)Remoteness	0.00	0.00	.966			0.00	.965		
**Step: Cohort**	0.18	0.20	<.001	0.018	<.001	.	.	.	.
**Model diagnostics**									
Significance (F-value)	86.64, p < .001								
R^2	28.5%								
Adjusted *R^2*	28.2%								

Note: Reported statistics are based on pooled results. With the exception of those for the remoteness predictor, beta coefficients displayed a high level of stability across models. The significant effect of cohort in predicting quality of life impairment once remoteness is accounted for (Model 1) suggests other (non-assessed) factors associated with cohort membership were also associated with quality of life outcomes. The univariate associations of indicators with psychological HRQoL impairment are also presented using Pearson's correlation coefficients (r); these correlations were consistent with the multivariate associations, suggesting that model design had little influence on the reported effects.

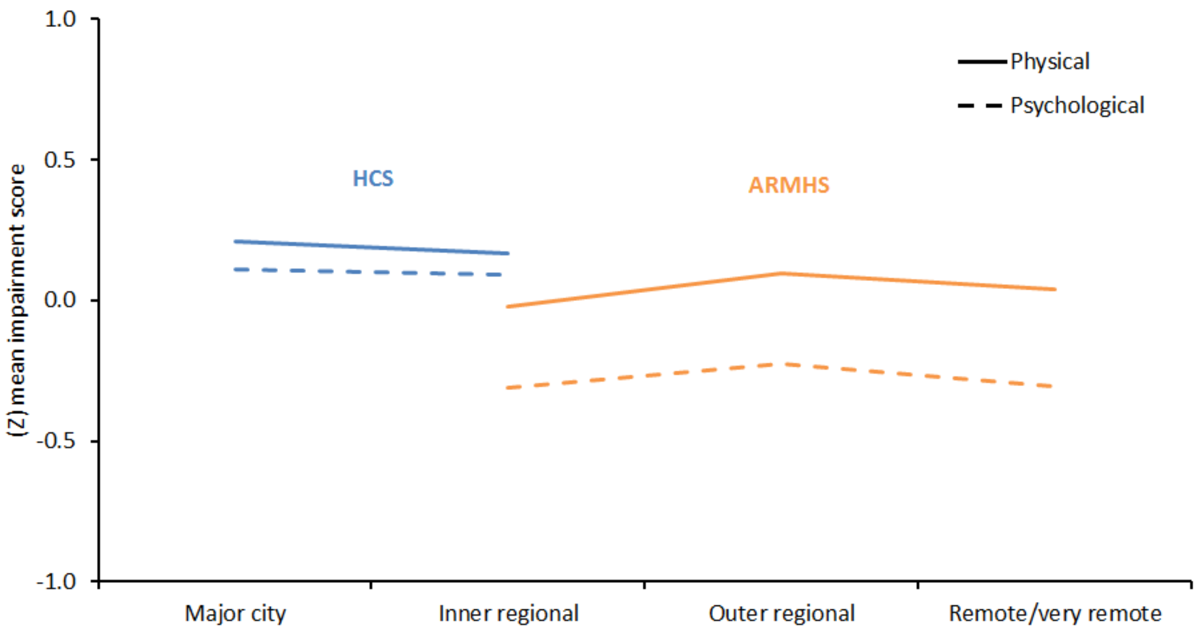

Figure S2. Standardized mean physical and psychological quality of life impairment scores by cohort and remoteness category. Note: HCS, Hunter Community Study; ARMHS, Australian Rural Mental Health Study.

The influence of cohort on the association of remoteness with physical and psychological HRQoL impairment (noted in Tables 2, S1 and S2) is reflected in Figure S2, which displays standardized mean impairment scores by remoteness category and cohort membership. Scores were standardized using normative means and standard deviations for the physical (M = 1.73, SD = 0.45) and psychological (M = 1.98, SD = 0.50) HRQoL domains reported in Allen et al. [1]. This plot shows physical impairment to be marginally higher in the HCS cohort. Psychological impairment was greater in the HCS compared to the ARMHS. Moreover, the slopes of the plot lines do not appear to vary by remoteness in either cohort, potentially raising concerns either about the sensitivity of the remoteness index, beyond the overall urban versus rural distinction, or the impact of other cohort related factors on HRQoL ratings.

1. Allen J, Inder K, Lewin T, Attia J, Kelly B: **Construct validity of the Assessment of Quality of Life - 6D (AQoL-6D) in community samples.** *Health Qual Life Outcomes* 2013, **11:**61.

Table S3. Sub-analysis: Hierarchical linear regression analysis of the correlates of physical and psychological quality of life impairment (replication of the primary analyses) – ARMHS sub analysis (N = 1176).

	Physical impairment					Psychological impairment				
	r	β	*p*	Step R^2	*p*	r	β	*p*	Step R^2	*p*
**Step: Demographic factors**				0.085	<.001				0.033	.000
(Z)Age	0.22	0.14	<.001			-0.06	-0.14	<.000		
Female	-0.05	-0.08	.004			0.07	0.04	.157		
12+ years education	-0.12	-0.10	.002			-0.04	-0.03	.333		
Married/de facto	-0.11	-0.08	.004			-0.10	-0.11	<.001		
Retired	0.21	0.14	<.001			0.08	0.12	<.001		
**Step: Cardiovascular & affective conditions**				0.080	<.001				0.123	<.001
Stroke	0.13	0.08	.005			0.10	0.09	.001		
Heart-attack/angina	0.21	0.16	<.001			0.07	0.08	.006		
Depression/Anxiety	0.21	0.22	<.001			0.35	0.33	<.001		
**Step: Other health indicators**				0.069	<.001				0.070	<.001
Diabetes	0.15	0.06	.019			0.05	0.03	.318		
Obesity	0.20	0.15	<.001			0.09	0.06	.028		
Current smoker	0.04	0.04	.118			0.10	0.04	.140		
High cholesterol	0.07	-0.03	.228			0.03	-0.04	.199		
Hypertension	0.17	0.07	.011			0.05	0.04	.172		
Social support	-0.20	-0.19	<.001			-0.31	-0.26	<.001		
(Z)Adverse life events	0.21	.	.			0.26	.	.		
(Z)Fin difficulty	0.34	.	.			0.30	.	.		
**Step: Contextual factors**				0.000	.516				0.003	.023
(Z)Social capital	-0.11	-0.02	.518			-0.21	-0.06	.023		
**Step: Interactions (2-way)**				0.000	.923				0.007	.018
(Z)Fin difficulty*(Z)Social capital	-0.04	.	.			-0.09	.	.		
Cardiovascular*(Z)Social capital	0.01	0.00	.874			0.01	0.00	.955		
Cardiovascular*Depression	0.03	0.02	.508			0.07	0.06	.055		
(Z)Social capital*Depression	0.01	0.00	.895			-0.07	-0.08	.012		
**Step: Interaction (3-way)**				0.001	.340				0.000	.796
Cardiovascular*Depression* (Z)Social capital	-0.03	-0.03	.341			-0.01	-0.01	.796		
**Model diagnostics**										
Significance (F-value)	18.62, p < .001					18.78, p < .001				
R^2	23.4%					23.6%				
Adjusted R^2	22.2%					22.3%				

Note: Reported statistics are based on pooled results (across multiple imputation datasets); this table presents results from models that, with the substitution of social capital for remoteness, include the same independent variables as the primary xTEND analyses. The univariate associations of indicators with HRQoL impairment are also presented using Pearson's correlation coefficients (r); with the possible exceptions of life time diagnoses of diabetes and hypertension with physical HRQoL impairment, and the influence of social capital on both physical and psychological HRQoL impairment, these correlations were consistent with the multivariate associations, suggesting model design had limited influence on the reported effects.
